# Development of a Patient Reported Measure of Experimental Transplants with HIV and Ethics in the United States (PROMETHEUS)

**DOI:** 10.1186/s41687-021-00297-y

**Published:** 2021-03-18

**Authors:** Shanti Seaman, Diane Brown, Ann Eno, Sile Yu, Allan B. Massie, Aaron A. R. Tobian, Christine M. Durand, Dorry L. Segev, Albert W. Wu, Jeremy Sugarman

**Affiliations:** 1 Department of Medicine, Johns Hopkins University School of Medicine, Baltimore, MD USA; 2 Department of Surgery, Johns Hopkins University School of Medicine, Baltimore, MD USA; 3 Department of Epidemiology, Johns Hopkins School of Public Health, Baltimore, MD USA; 4 Department of Pathology, Johns Hopkins University School of Medicine, Baltimore, MD USA; 5 Department of Health Policy and Management, Johns Hopkins Bloomberg School of Public Health, Baltimore, MD USA; 6 Berman Institute of Bioethics, Johns Hopkins University, 1809 Ashland Ave, Baltimore, MD 21205 USA

**Keywords:** Acquired immunodeficiency syndrome, Care delivery, Ethics, Organ procurement and transplantation, Patient-reported experiences, Patient safety, Quality of care

## Abstract

**Background:**

Transplantation of HIV-positive (HIV+) donor organs for HIV+ recipients (HIV D+/R+) is now being performed as research in the United States, but raises ethical concerns. While patient-reported outcome measures are increasingly used to evaluate clinical interventions, there is no published measure to aptly capture patients’ experiences in the unique context of experimental HIV D+/R+ transplantation. Therefore, we developed PROMETHEUS (**p**atient-**r**eported **m**easure of **e**xperimental **t**ransplants with **H**IV and **e**thics in the **U**nited **S**tates). To do so, we created a conceptual framework, drafted a pilot battery using existing and new measures related to this context, and refined it based on cognitive and pilot testing. PROMETHEUS was administered 6-months post-transplant in a clinical trial evaluating these transplants. We analyzed data from the first 20 patient-participants for reliability and validity by calculating Cronbach’s alpha and reviewing item performance characteristics.

**Results:**

PROMETHEUS 1.0 consisted of 29 items with 5 putative subscales: Emotions; Trust; Decision Making; Transplant; and Decision Satisfaction. Overall, responses were positive. Cronbach’s alpha was > 0.8 for all subscales except Transplant, which was 0.38. Two Transplant subscale items were removed due to poor reliability and construct validity.

**Conclusions:**

We developed PROMETHEUS to systematically capture patient-reported experiences with this novel experimental transplantation program, nested it in an actual clinical trial, and obtained preliminary data regarding its performance.

**Supplementary Information:**

The online version contains supplementary material available at 10.1186/s41687-021-00297-y.

## Background

People living with HIV (PLWH) are at increased risk of organ failure that is amenable to treatment with organ transplantation. However, transplant candidates living with HIV experience longer organ wait times compared to their HIV-negative (HIV-) counterparts, resulting in increased waitlist mortality [1, 2]. In effort to ameliorate this inequity, as well as to alleviate the organ shortage in general, novel transplantation of HIV-positive (HIV+) donor organs for HIV+ recipients (HIV D+/R+) is being performed [3, 4]. While early experience suggests that these transplants are safe and effective [5, 6], HIV D+/R+ transplantation raises ethical issues that must be addressed [7]. For example, there are uncertain medical risks, such as those associated with the use of immunosuppressive agents along with antiretroviral therapy and of acquiring a second strain of HIV leading to HIV-superinfection [8, 9]. There are also psychosocial risks related to having to choose between accepting an HIV+ donor organ for which there are limited safety and efficacy data versus the burden of staying on an organ waitlist awaiting standard transplantation with one that is HIV- [7]. Accordingly, to help assess the overall appropriateness of novel HIV D+/R+ organ transplantation, it is essential to ascertain the perspectives of patients undergoing these transplants.

There is increasing recognition of the importance of using patient-reported outcome measures (PROMs) and patient-reported experience measures (PREMs) to assess the effects of interventions being evaluated in research [10]. Similarly, PROMs and PREMs are being applied in clinical practice [11–14]. In the field of transplantation, there are existing, patient-reported measures of the informed consent process, emotions, decisional regret and trust [15–20]. However, these measures may not apply to the unique context of experimental HIV D+/R+ transplantation and do not capture the range of ethical issues raised by this novel procedure.

Therefore, we aimed to create PROMETHEUS: a **p**atient-**r**eported **m**easure of **e**xperimental **t**ransplants with **H**IV and **e**thics in the **U**nited **S**tates. This article describes the development of PROMETHEUS and its preliminary performance in a large-scale national clinical trial of HIV D+/R+ renal transplantation funded by the U.S. National Institutes of Health. By including PROMETHEUS in this and other related transplant settings, we hope to facilitate a more comprehensive analysis of HIV D+/R+ transplantation. While the primary outcomes of this trial are clinical, the impact of these novel transplants on the quality of life of the patients receiving them and whether ethical issues are encountered are also critical. Without a focused assessment that captures the unique context of novel HIV D+/R+ transplantation, the rights and interests of the patients this practice intends to benefit are at risk of being overshadowed by accelerating clinical progress while the window of opportunity to implement corrected policies and practices in a systematic manner closes.

## Methods

PROMETHEUS was developed as part of a larger multidisciplinary project aimed at understanding the ethical, legal and social issues associated with HIV D+/R+ transplantation. The larger project includes in-depth interviews with recipients of HIV D+/R+ transplants, potential HIV+ living donors [21] and independent recipient advocates [22] as well as a quantitative survey of transplant candidates living with HIV on the organ transplant waitlist regarding their willingness to accept HIV D+/R+ transplants [23].

**Fig. 1:**
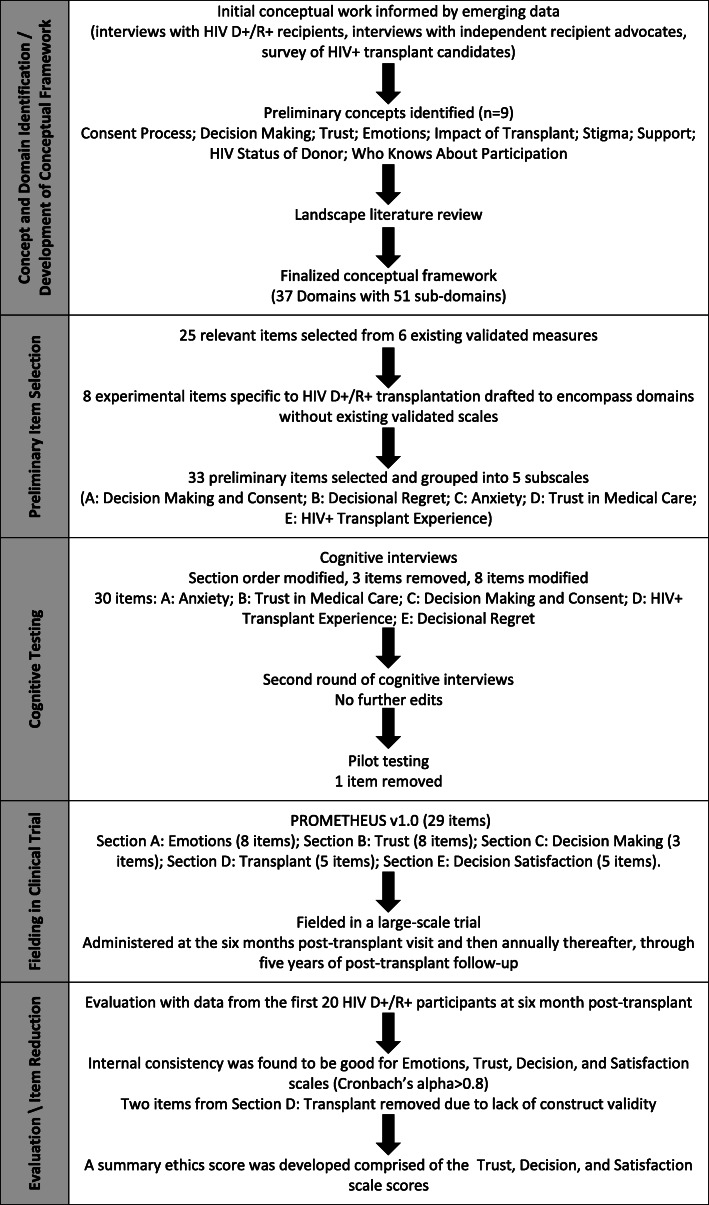
Overview of the process of developing and testing PROMETHEUS

Work to develop and test PROMETHEUS was approved by the Johns Hopkins Medicine Institutional Review Board. An overview of the development process is displayed in Fig. 1.

### Concept and domain identification

The project built on early conceptual work regarding ethical issues in HIV D+/R+ transplantation by members of our project team [7]. Specifically, emerging data from our interview and survey studies informed the development of a more comprehensive conceptual framework with relevant domains to be included in PROMETHEUS. In particular, preliminary thematic analyses of interviews with HIV D+/R+ transplant recipients at Johns Hopkins Hospital along with survey responses of transplant candidates living with HIV from nine transplant centers in the United States were incorporated into the conceptual framework. A landscape review of the literature was then conducted using PubMed to identify other potentially relevant issues reported by patients undergoing organ transplantation, experimental procedures in general, or who were in other similar clinical contexts. Items that had been used to collect patient-reported outcomes and experiences in these contexts were also ascertained as part of the literature review.

### Item selection, development and cognitive testing

An initial pool of items was created by identifying the domains in our conceptual framework for which there were existing published measures and then those measures were reviewed for relevant items. New items specific to HIV D+/R+ transplantation were drafted to capture concepts and domains without existing validated scales.

Cognitive interviews regarding an initial pool of candidate items were conducted with a convenience sample of PLWH who receive medical care at the Johns Hopkins Medical Institutions. Evaluation for organ transplantation was not an inclusion criterion for these interviews, given the relatively limited patient population. At the time of the cognitive interviews, there were also too few recipients of HIV D+/R+ transplants to create an adequate sample of PLWH who had actually received a HIV D+/R+ transplant. To account for this, participants were asked to imagine that they had kidney failure and were presented with a detailed description of how kidney failure might impact their daily lives.

*“Imagine that you had kidney failure. You often felt tired and sluggish. Sometimes you felt swollen up, dizzy, or out of breath. A piece of tubing had been inserted into your chest. This restricted some of your physical movement and was sometimes uncomfortable. Three times per week you had to go to the dialysis center and spend about 6 hours hooked up to a dialysis machine. You followed a strict diet: low salt, little meat, and small amounts of fluid. You decided to have a kidney transplant if a matched kidney could be found for you. After waiting for some time on the transplant list, you decided to accept an HIV+ kidney instead of waiting for an HIV- kidney so that you could get off dialysis and return to a more normal life. You had transplant surgery and got a new HIV+ kidney. You now feel pretty much how you felt before you had kidney failure, when your kidneys were still working.”*

In addition, participants were informed that HIV D+/R+ transplants were experimental and that the survey being developed was going to be used in a study assessing them. Following written consent, participants were asked to evaluate individual survey items by providing their thoughts, opinions, and understanding of each question using standard cognitive interview techniques [24]. The interviewer took notes and aggregated interview responses to write narrative accounts summarizing the cognitive interviews. The interviews lasted approximately 30 min and participants were given a $25 gift card as compensation for their time.

The results of the first round of cognitive interviews were analyzed and survey items were revised to address issues raised by participants. A second round of cognitive interviews with the revised items was conducted with a new convenience sample of PLWH.

### Pilot testing

For pilot testing, participants were again asked to imagine they had kidney failure along with a written (in contrast to the oral description used in the cognitive interviews) description of how kidney failure might impact their daily lives. Due to the limited population of patients being evaluated for these transplants, pilot testing was then conducted with a new convenience sample of PLWH who were not being evaluated for organ transplantation at the time of interview. As in the cognitive interviews, participants were informed that HIV D+/R+ transplants were experimental and that the survey being developed was going to be used in a study assessing them. Participants provided written consent. Surveys took approximately 15 min to complete and participants were given a $25 gift card as compensation for their time. Minimal revisions to survey items were made as a result of pilot testing.

### Preliminary assessment of reliability

Using the data from the pilot testing, a preliminary assessment of the reliability of the putative scales and the overall battery was conducted by calculating Cronbach’s alpha using Stata 16.1. These results were used to finalize PROMETHEUS Version 1.0.

### Fielding in a clinical trial

PROMETHEUS Version 1.0 is currently being fielded in a large-scale national study funded by the U.S. National Institutes of Health, which began enrollment in July 2018 (U01AI134591: HOPE in Action: A Multicenter Clinical Trial of HIV-to-HIV Deceased Donor Kidney Transplantation [NCT03500315]). In this study, PROMETHEUS is administered beginning at the 6 months post-transplant visit and then annually thereafter, through 5 years of post-transplant follow-up. The study also incorporates standard measures of health related quality of life (KDQOL-SF™ v1.3 and RAND SF-20, respectively) [25, 26] to supplement measures of ethics and related experiences being elicited by PROMETHEUS. The analysis included in this paper only includes the 6-month post-transplant responses of the first 20 participants who received an HIV D+ organ in the study.

### Internal consistency reliability, construct validity and item reduction

Using the data from the initial participants in the study, an analysis of PROMETHEUS Version 1.0 was conducted to assess reliability and validity. Internal consistency reliability was estimated using Cronbach’s alpha. A Cronbach’s alpha of 0.7 or greater was considered acceptable [27] and item-total correlations were calculated to determine the extent to which items within the same scale measure the same construct. Construct validity was explored by examining convergent and discriminant construct validity using a multitrait-multimethod matrix. Score distributions were calculated for each item and subscale score to further examine construct validity, including mean, median, and the proportion of minimum and maximum responses to assess the floor and ceiling effects. Construct validity was further tested using the physical and mental component summary (PCS/MCS) scores of the KDQOL-36™ [25]. Items that lacked construct validity were removed.

## Results

### Concept and domain identification

Based on the preliminary content analyses of interviews with transplant recipients of HIV+ donor organs and surveys of PLWH on transplant waitlists, the following key ethics concepts were identified for inclusion in our preliminary conceptual framework: consent process, decision making, trust, emotions, impact of transplant, stigma, support, HIV status of donor, and who knows about study participation. These 9 preliminary concepts were interrogated in our landscape literature review in PubMed. Using 8 search terms in February 2017, 412 postings were identified and narrowed to 15 relevant articles based on review of titles and abstracts as well as the full article if necessary. The search term “decisional regret” and “surgery” resulted in 62 postings of which 4 were determined to be of relevance [28–31]; “consent” and “satisfaction” and “transplant” resulted in 78 postings of which 1 was determined to be of relevance [17]; “decision regret” [title] resulted in 27 postings of which 2 were determined to be of relevance [32, 33]; “decisional regret” [title] resulted in 14 postings of which 1 was determined to be of relevance [34]; “satisfaction” [title] and “consent” [title] resulted in 18 postings of which 1 was determined to be of relevance [35]; “evaluation of consent process” [title] resulted in 30 postings of which 1 was determined to be of relevance [36]; “managing expectations” and “transplant” resulted in 18 postings of which 3 were determined to be of relevance [16, 19, 20]; and “kidney transplant” and “expectations” resulted in 165 postings of which 2 were determined to be of relevance [15, 18]. In addition to the 15 articles identified through these PubMed searches, 4 articles known to members of the study team were included for review [37–40]. Together, these 19 articles described 13 tools used to collect patient-reported outcomes and experiences (Table 1).

**Table 1:** PROMETHEUS literature review summary

Concept	Tool(s) Used	Quantitative or Qualitative	Clinical Context	Key Points	Reference
Consent Process	Spielberger STAI^a^, Questionnaire	Quantitative	Cardiac Electrophysiology	Patients prefer to be made aware of possible risks. No significant difference between oral, written, and video consent formats.	Goldberger et al. 2011 [35]
	EDICT^a^	Quantitative	Liver Transplantation	Development of “Evaluation of Donor Informed Consent Tool” to assess consent comprehension.	Gordon et al. 2015 [29]
	Narrative interviews	Qualitative	Bone-marrow Transplantation	Patients’ views toward consent process change after reporting disappointment with procedure.	Little et al. 2008 [17]
	Telephone questionnaire	Qualitative	Open Inguinal Hernia Repairs	Patients have better recall of benefits than of complications, intraoperative details, or post-operative instructions.	Uzzaman et al. 2012 [36]
	QuIC^a^	Quantitative	Oncology	Therapeutic misconceptions held by participants and investigators need to be directly addressed to improve the quality of informed consent.	Joffe et al. 2001 [38]
	Deliberative Engagement Sessions	Qualitative	General Medical Research	Patients want to be told about research and have a choice but are open to streamlining those disclosures.	Kass et al. 2016 [39]
	BICEP^a^	Qualitative	General Medical Research	Independent telephone evaluations of the quality of informed consent are feasible. More focus needed on voluntariness of continued participation and that participation has purpose aside from personal benefit.	Sugarman et al. 2005 [40]
Decision Making	EORTC QLQ-C30^a^, DRS^a^	Quantitative	Oncology	3 types of medical decision-making roles: passive, collaborative, and active. Time since treatment may have greatest effect on decisional regret.	Davison et al. 2003 [28]
	CARE^a^, SDM-Q-9^a^, CPS/PPS^a^, DRS^a^	Quantitative	Oncology	A patient’s desired role in the decision-making process must be assessed.	Nicolai et al. 2016 [33]
	Systematic review	Quantitative	Health Decisions	Less decisional conflict, greater satisfaction with information provided and more involvement in the decision-making process were associated with less regret.	Becerra Perez et al. 2016 [32]
	Qualitative data review	Qualitative	High-Risk Surgery in Older Adults	Discussion of treatment options, clarification of goals, and preparation for expected and unexpected outcomes are important for informed decision-making.	Steffens et al. 2016 [31]
	DRS^a^	Quantitative	Major Thoracic and Abdominal Surgery	Clinicians need to emphasize that the patient has a real choice. Correlation between depression and decisional regret.	Wilson et al. 2017 [34]
Trust	Systematic review, thematic synthesis	Qualitative	Renal Transplantation	Tenuous eligibility causes patients to feel unfairly dismissed and suspicious of discrimination.	Tong et al. 2015 [20]
	Questionnaire	Quantitative	General Medical Research	Components of trust (fidelity, honesty, safety, etc.) did not emerge as distinct factors. Patients did not differentiate trust in physician researchers from trust in medical researchers generally.	Hall et al. 2006 [37]
Emotions	EQ-5D QoL Instrument^a^	Quantitative	Renal Transplantation	Clinicians have more accurate perceptions of post-transplant quality of life than patients do. Patients may not fully understand what post-transplant treatment will look like.	Cleemput et al. 2003 [15]
	Cantril’s ladder^a^, VAS^a^	Quantitative	Renal Transplantation	Patients overestimate post-transplant quality of life. Low optimism found to be a risk factor for early distress.	Schulz et al. 2014 [18]
	Semi-structured interviews	Qualitative	Islet Cell Transplantation	Patients need to be prepared for the risks involved in transplant and need to develop coping strategies for disappointment at any stage.	Speight et al. 2016 [19]
	Systematic review, thematic synthesis	Qualitative	Renal Transplantation	Balancing concerns for their living donor along with their own health creates tension for patients. Major themes in patient attitudes towards living donor transplantion.	Hanson et al. 2015 [30]
Impact of Transplant	Self-report surveys, Diary, Medication Monitoring	Qualitative	Renal Transplantation	Patients had unrealistic expectations of return to normalcy and were not prepared for the challenges of living after transplantation.	Crawford et al. 2017 [16]

^a^*Explanation of Tools:*

Spielberger STAI: Spielberger State-Trait Anxiety Inventory – 20 questions that assess anxiety at one particular moment in time (state) and another 20 questions that assess general anxiety level (trait)

EDICT: Evaluation of Donor Informed Consent Tool – 49 true/false/unsure questions designed to assess living donors' comprehension of the living donation process.

QuIC: Quality of Informed Consent – 34 item questionnaire to assess the informed consent process for clinical research of cancer therapies. Responses to individual questions are combined to create a knowledge score ranging from 0 (least) to 100.

BICEP: Brief Informed Consent Evaluation Protocol – short telephone interview to evaluate the informed consent process used to create an Informed Consent Aggregate Score (ICAS) and Therapeutic Misconception Aggregate Score (TMAS). Each item is scored 1 for Yes and 0 for No and the aggregate score is the sum.

EORTC QLQ-C30: European Organization for Research and Treatment of Cancer Quality of Life Questionnaire (version 3) – a 30-item questionnaire grouped into five functional domains (physical, role, cognitive, emotional, social), three symptom domains (fatigue, pain, nausea and vomiting), five single-symptom items (dyspnea, insomnia, anorexia, diarrhea, constipation), a financial concerns item, an overall health domain, and an overall quality of life domain.

DRS: Five-item self-reported Likert Scale (4, strongly disagree; 3, disagree; 2, neither agree nor disagree; 1, agree; and 0, strongly agree) including: 1, it was the right decision; 2, I regret the decision that was made; 3, I would make the same decision if I had to do it again; 4, the decision did me a lot of harm; and 5, the decision was a wise one.

CARE: Consultation and Relational Empathy measure – 10 items that are rated on a five-point Likert scale to assess patients’ views of physicians’ empathic communication skills.

SDM-Q-9: Nine-item Shared Decision-Making Questionnaire that asks patients to rate items on a six-point Likert scale. Items cover: 1, disclosure that a decision must be made; 2, formulation of the partners’ equality; 3, presentation of treatment options; 5, investigation of the patient’s understanding and expectations; 6, identification of both parties’ preferences; 7, negotiation; 8, arriving at a shared decision; and 9, arranging follow-up.

CPS: Control Preferences Scale – asks the patient which of five options best describes their preferred role in decision-making.

PPS: Patient Perception Scale – asks the patient which of five options best describes how the decision was made.

EQ-5D QoL Instrument: Measures health-related quality of life using descriptive and valuation parts. The descriptive part is made up of five items that represent different dimensions of health (mobility, self-care, usual activities, pain and discomfort, and anxiety and depression) along with three levels of severity (no problems, some problems, and severe problems). The valuation part consists of a Visual Analogue Scale (VAS) where respondents rank their quality of life for each health state described in the descriptive part from 0 (worst imaginable health state) to 100 (best imaginable health state).

Cantril’s ladder: Single-item measure of quality of life that is easy to understand and easy to administer.

VAS: A 10-point visual analogue scale ranging from 1 (worst imaginable quality of life) to 10 (best imaginable quality of life)

Based on this literature review and the preliminary analyses of our interview and survey studies, 37 domains and 51 sub-domains were identified that comprised our final conceptual framework for PROMETHEUS (Table 2).

**Table 2:** PROMETHEUS concepts and domains with items

Concept	Domain	Sub-domains		Borrowed Scale	Preliminary Item	Final Item in PROMETHEUS v1.0
**Consent Process**	**Satisfaction**	With process		BICEP	7. Were you satisfied with the informed consent process?	C3. I was satisfied with the consent process to get the new organ.
		With information given				
	**Information Provided**	Sufficiency		BICEP	1. Did you get all the information you needed to make a good decision about participating in [study]?	C1. I got all the information I needed to make a good decision about getting the new organ.
		Complexity				
		Understood				
	Questions	Asked questions		–	–	–
		All answered				
	**Vulnerability**	Illness		–	–	–
		Time		–	–	–
		Pressure		BICEP	3. Did you feel any pressure to participate in [study]?	C2. I felt pressure to accept the new organ.
				*Experimental Item*	3. When I was told there was an organ available for me I felt pressure to accept it.	*REMOVED*
**Decision Making**	**Reasons to Accept HIV D+ Organ**	Already HIV+		–	–	–
		Avoid/stop dialysis		–	–	–
		Shorter wait time, healthier at time of transplant		–	–	–
		More likely to get transplant		*Experimental Item*	5. Because of my race, joining this study was the best chance I had at getting a transplant.	D3. Because of my race, accepting this organ was the best chance I had at getting a transplant.
				*Experimental Item*	6. Because of my HIV status, joining this study was the best chance I had at getting a transplant.	D4. Because of my HIV status, accepting this organ was the best chance I had at getting a transplant.
		Contribution to science / research, help others		–	–	–
	**Discuss/Consult Study Participation With**	Partner/Spouse		–	–	–
		Family		–	–	–
		Friends		–	–	–
		Study doctor(s)		*Experimental Item*	2. Trust in the healthcare workers on the study team was a significant factor in my decision to participate.	D2. Trust in doctors and nurses who took care of me was a significant factor in my decision to get a new organ.
		Other doctor(s)		–	–	–
	Concerns	Transplant concerns		–	–	–
		HIV concerns				
		Other concerns				
	Role	Passive		–	–	–
		Collaborative				
		Active				
	Time to Decide	Immediate or almost immediate		–	–	–
		Some time				
**Trust**	**In Medicine**	–		A-WFPT Scale	1. Sometimes doctors care more about what is convenient for them than about their patients’ medical needs.	B1. Sometimes doctors care more about what is convenient for them than about their patients’ medical needs.
				A-WFPT Scale	2. Doctors are extremely thorough and careful.	B2. Doctors are extremely thorough and careful.
				A-WFPT Scale	3. You completely trust doctors’ decisions about which medical treatments are best.	B3. You completely trust doctors’ decisions about which medical treatments are best.
				A-WFPT Scale	4. A doctor would never mislead you about anything.	B4. A doctor would never mislead you about anything.
				A-WFPT Scale	5. All in all, you trust doctors completely.	B5. All in all, you trust doctors completely.
				R-HCSD Scale	1. The Health Care System does its best to make patients’ health better.	B7. The Health Care System does its best to make patients’ health better.
	**In Medical Research**	–		R-HCSD Scale	9. The Health Care System experiments on patients without them knowing.	B6. The health care system experiments on patients without them knowing.
				*Experimental Item*	2. Trust in the healthcare workers on the study team was a significant factor in my decision to participate.	D2. Trust in doctors and nurses who took care of me was a significant factor in my decision to get a new organ.
	**In Institutions**	–		MTHCS Scale	16. Health care institutions provide the highest quality in medical care.	*REMOVED*
**Emotions**	**Decisional Regret**	No regrets		Decision Regret Scale (DRS)	1. It was the right decision.	E1. It was the right decision.
				DRS	3. I would go for the same choice if I had to do it over again.	E3. I would make the same decision if I had to do it again.
				DRS	5. The decision was a wise one.	E5. The decision was a wise one.
		Initial regrets		–	–	–
		Lingering regrets		DRS	2. I regret the choice that was made.	E2. I regret the decision that was made.
				DRS	4. The choice did me a lot of harm.	E4. The decision did me a lot of harm.
	**Feeling Unprepared for Transplant**	At time of tx	Time b/w consent & tx very short	–	–	–
			Not ready for tx when first offered	–	–	–
		For recovery	Immunosuppression	*Experimental Item*	1. My recovery was harder than I imagined.	D1. My recovery was harder than I imagined.
			Follow-up visits			
			Generally worse than imagined			
	Grateful/Lucky	Thanks to donor/donor family		–	–	–
		Thanks to medical team				
		Thanks to research				
		Thanks to “God”/religion				
	**Fear**	–		PROMIS Anxiety SF 8a	1. I felt fearful …	A1. I felt fearful …
	Fairness	Self		–	–	–
		Others				
**Impact of Transplant**	Physical Function	More active, life back to normal		–	–	–
		Less active, healthier at/before transplant				
	**Social Function**	Relationships w/partner, family, friends		PROMIS Anxiety SF 8a	2. I found it hard to focus on anything other than my anxiety …	A2. I found it hard to focus on anything other than my anxiety …
		Ability to work				
		Ability to travel				
	Overall Health	–		–	–	–
	**Mental Health/ Cognitive Abilities**	–		PROMIS Anxiety SF 8a	1. I felt fearful …	A1. I felt fearful …
				PROMIS Anxiety SF 8a	2. I found it hard to focus on anything other than my anxiety …	A2. I found it hard to focus on anything other than my anxiety …
				PROMIS Anxiety SF 8a	3. My worries overwhelmed me …	A3. My worries overwhelmed me …
				PROMIS Anxiety SF 8a	4. I felt uneasy …	A4. I felt uneasy …
				PROMIS Anxiety SF 8a	5. I felt nervous …	A5. I felt nervous …
				PROMIS Anxiety SF 8a	6. I felt like I needed help for my anxiety …	A6. I felt like I needed help for my anxiety …
				PROMIS Anxiety SF 8a	7. I felt anxious …	A7. I felt anxious …
				PROMIS Anxiety SF 8a	8. I felt tense …	A8. I felt tense …
**Stigma**	**HIV Status**	–		*Experimental Item*	6. Because of my HIV status, joining this study was the best chance I had at getting a transplant.	D4. Because of my HIV status, accepting this organ was the best chance I had at getting a transplant.
				*Experimental Item*	7. Being a part of this study made me feel better about having HIV.	D5. Being able to get this transplant made me feel better about having HIV.
				*Experimental Item*	8. This study makes me feel like my HIV diagnosis gives me the unique ability to help others.	*REMOVED*
	Addiction / Substance Abuse	–		–	–	–
	**Race/Ethnicity**	–		R-HCSD Scale	7. Patients get the same medical treatment from the Health Care System, no matter what the patient’s race or ethnicity.	B8. Patients get the same medical treatment from the health care system, no matter what the patient’s race or ethnicity.
				*Experimental Item*	5. Because of my race, joining this study was the best chance I had at getting a transplant.	D3. Because of my race, accepting this organ was the best chance I had at getting a transplant.
	Poverty	–		–	–	–
	Sexuality	–		–	–	–
	Gender Identity	–		–	–	–
	Transplant/Illness	–		–	–	–
**Support**	From Partner/Spouse	–		–	–	–
	From Family	–		–	–	–
	From Friends	–		–	–	–
	From Medical Team	–		*Experimental Item*	2. Trust in the healthcare workers on the study team was a significant factor in my decision to participate.	D2. Trust in doctors and nurses who took care of me was a significant factor in my decision to get a new organ.
	From Research Team	–		*Experimental Item*	2. Trust in the healthcare workers on the study team was a significant factor in my decision to participate.	D2. Trust in doctors and nurses who took care of me was a significant factor in my decision to get a new organ.
**HIV Status of Donor**	**False-Positive or HIV- Donor is Better**	–		*Experimental Item*	4. I was relieved to learn that I got an HIV negative organ and not one that was HIV positive.	*REMOVED*
	Does Not Matter			–	–	–
Who Knows About Study Participation	Want to Tell/Has Told People	–		–	–	–
	Does Not Want to Tell	HIV status not public		–	–	–
		Don’t want others reactions/ opinions (negative or positive)		–	–	–
		Privacy		–	–	–
		Worried about stigma		–	–	–

### Item selection, development and cognitive testing

Domains from our conceptual framework that had existing validated measures were identified and reviewed for potential inclusion in PROMETHEUS. Six such scales were considered: the “Brief Informed Consent Evaluation Protocol” [40]; the “Decision Regret Scale” [41]; the “PROMIS Item Bank v1.0 – Emotional Distress – Anxiety – Short Form 8a” [42]; the “Abbreviated Wake Forest Physician Trust Scale – Patient trust in the medical profession^”^ [43]; the “Revised Health Care System Distrust Scale” [44]; and the “Multidimensional Trust in Health Care Systems Scale” [45].

An initial pool of 25 relevant items was selected from these 6 existing scales. Three out of 12 items were selected from the “Brief Informed Consent Evaluation Protocol” (BICEP) [40] to cover consent process domains. All 5 items from the “Decision Regret Scale” [41] were selected to measure decisional regret. All 8 items from the “PROMIS Item Bank v1.0 – Emotional Distress – Anxiety – Short Form 8a” (PROMIS Anxiety SF 8a) [42] were selected to cover impact of transplant and emotions domains. All 5 items from the “Abbreviated Wake Forest Physician Trust Scale – Patient trust in the medical profession” (A-WFPT Scale) [43] were selected to cover trust. Three out of 9 items were selected from the “Revised Health Care System Distrust Scale” (R-HCSD Scale) [44] to cover trust and stigma domains. Finally, 1 out of 17 items were selected from the “Multidimensional Trust in Health Care Systems Scale” (MTHCS Scale) [45] to cover the domain of trust in institutions. These 25 questions encompass 11 out of 37 domains and 5 out of 9 concepts from our conceptual framework (Table 2).

The following 10 key domains were then identified as not having existing validated scales: vulnerability, reasons to accept an HIV D+ organ, discuss/consult study participation, trust in medical research in the context of these transplants, feeling unprepared for transplant, stigma related to HIV status, stigma related to race/ethnicity, support from the medical team, support from the research team, and the perception that a false-positive (i.e., a donor organ that was initially assumed to be from an HIV+ donor based on initial testing at the time a transplant decision was being made, but was subsequently found not to be HIV+) or HIV- organ is better than an HIV+ organ. Accordingly, 8 experimental items specific to HIV D+/R+ transplantation were drafted, resulting in a preliminary pool of 33 items. This preliminary pool of 33 items was then grouped into 5 subscales: A – Decision Making and Consent, B – Decisional Regret, C – Anxiety, D – Trust in Medical Care, and E – HIV+ Transplant Experience (our experimental items). In doing so, the project team reached consensus that all essential aspects of our conceptual framework for PROMETHEUS were addressed by these items.

In the first round of cognitive interviews with 7 PLWH, all participants self-identified as Black/African American and 4 self-identified as female. Two participants were 35–44 years old, 2 were 45–54 years old, and 3 were 55–64 years old. Two participants had less than a high school education, 4 graduated from high school or received their Graduate Equivalent Degree, and 1 had a bachelor’s degree.

As a result of these interviews, the orderings of the subscales were adjusted, some items were modified and others deleted. Specifically subscale A – “Decision Making and Consent” was moved to be the third subscale and all 3 questions were changed to statements rather than questions so that the response options could be on a 5 point Likert-type scale from Never to Always. Subscale B – “Decisional Regret” was moved to be the last subscale. Subscale C – “Anxiety” was moved to be the first subscale. Subscale D – “Trust in Medical Care” was moved to be the second subscale and the question from the MTHCS Scale (“Health care institutions provide the highest quality in medical care”) was removed due to respondent confusion regarding what a health care institution was and their uncertainty regarding how to interpret “highest quality in medical care.” Despite removing this item, the concept of trust seemed to be sufficiently covered by the domains trust in medicine and trust in medical research.

For Subscale E – “HIV+ Transplant Experience”, 2 out of 8 items were removed and wording changes were made to 5 items. The HIV+ Transplant Experience subscale was also moved to be the penultimate subscale. The fourth item, “I was relieved to learn that I got an HIV negative organ and not one that was HIV positive” was removed because all but one respondent indicated that they strongly agree because taking an HIV+ donor organ is a risk, makes transplantation complicated, and might not work as well. The item, “This study makes me feel like my HIV diagnosis gives me the unique ability to help others” was removed because it was unclear and the domain of stigma due to HIV status was adequately captured by 2 other items (“Because of my HIV status, joining this study was the best chance I had at getting a transplant” and “Being a part of this study made me feel better about having HIV”, respectively). The item, “Trust in the healthcare workers on the study team was a significant factor in my decision to participate” was modified to, “Trust in doctors and nurses who took care of me was a significant factor in my decision to get a new organ” due to respondents’ inaccurate belief that “study team” meant people studying to become doctors. Another was simplified from, “When I was told there was an organ available for me I felt pressure to accept it” to, “When I was told there was an organ for me, I felt pressure to accept it”. Two other items (“Because of my race, joining this study was the best chance I had at getting a transplant”; and “Because of my HIV status, joining this study was the best chance I had at getting a transplant”, respectively) were modified to, “Because of my race, accepting this organ was the best chance I had at getting a transplant”; and “Because of my HIV status, accepting this organ was the best chance I had at getting a transplant” to clarify what “joining this study” meant. “Being a part of this study made me feel better about having HIV” was also modified to, “Being able to get this transplant made me feel better about having HIV” to omit confusion over what “this study” was referring to.

As a result of these changes, 30 items were included in the following subscales: Section A – Anxiety, Section B – Trust in Medical Care, Section C – Decision Making and Consent, Section D – HIV+ Transplant Experience, Section E – Decisional Regret.

A second round of cognitive interviews was conducted with 5 PLWH to get feedback on the revised 30 items and their ordering. Four participants self-identified as Black/African American and 1 self-identified as White, Black, and Puerto Rican. One out of 5 participants self-identified as female, 1 was 45–54 years old, and the remaining 4 were 55–64 years old. Three participants had less than a high school education, and the remaining 2 had graduated from high school or received their Graduate Equivalent Degree. Analysis of the second round of cognitive interviews found that no further edits to survey items were needed prior to pilot testing.

The 30 item pilot survey was then administered to 20 participants. Seventeen self-identified as Black/African American and 3 self-identified as American Indian/Alaska Native. Eleven participants were female, 2 were 25–34 years old, 4 were 35–44 years old, 6 were 45–54 years old, and 8 were 55–64 years old. Seven participants had less than a high school education, 10 graduated from high school or received their Graduate Equivalent Degree, 2 had a bachelor’s degree, and 1 had a graduate degree. As a result of pilot testing, 1 item was removed from the HIV+ Transplant Experience subscale (“When I was told there was an organ for me, I felt pressure to accept it”) since the domain of vulnerability during the consent process was adequately captured by an item in the Decision Making subscale (“I felt pressure to accept the new organ”).

### Initial reliability testing

Cronbach’s alpha for all items was 0.89. For the putative subscales this value was: Emotions = 0.93; Trust = 0.82; Decision Making = 0.57; Transplant = 0.59; and Decision Satisfaction = 0.71. Based on this performance in a hypothetical setting, no further refinements were made to the battery.

### Fielding in a clinical trial

The final battery (See Additional file 1: PROMETHEUS Fielded Battery) was then prepared for fielding in the national trial described above. A cover page included the following instructions: “This survey includes a wide variety of questions related to how you are feeling and the decisions you made regarding the possibility of receiving an HIV+ donor organ” and, “Directions: Please circle the one response that BEST describes your answer to the question”. The 29 item PROMETHEUS Version 1.0 consisted of 5 subscales: Section A – Emotions (8 items), Section B –Trust (8 items); Section C – Decision Making (3 items); Section D – Transplant (5 items), Section E – Decision Satisfaction (5 items). Section A – Emotions (rather than being termed Anxiety to be more neutral) gave instruction to consider the past 7 days only (“In the past 7 days …” ) and response options were given as a 5 point Likert-type scale from Never to Always. The rest of the sections gave response options as a 5 point Likert scale from Strongly Agree to Strongly Disagree. Section E – Decision Satisfaction, instructed that, “The next set of questions are about your decision to get the new organ”. A section to be completed by the study team at end of the battery queried how PROMETHEUS was completed (“Self-administered by patient” vs. “In-person interview” vs. “Telephone interview” vs. “Other; specify”) and if it was completed by direct data entry.

### Evaluation in the target population and item reduction

The first 20 trial participants had a median age of 53 years (IQR: 45–58); 20% were female; 75% identified as Black/African American and 10% identified as Asian; none identified as Latino/Hispanic (Table 3).

**Table 3:** Characteristics of transplant trial participants

Characteristics	*N* = 20
Age at transplant, median years (IQR)	53 (45, 58)
Female sex	4 (20%)
Race	
White	3 (15%)
Black/African American	15 (75%)
Asian	2 (10%)
Ethnicity	
Hispanic or Latino	0 (0%)
Not Hispanic or Latino	20 (100%)

Internal consistency was acceptable for the Emotions, Trust, Decision, and Satisfaction scales (Cronbach’s alpha > 0.8) (Table 4). The 5 HIV+ Transplant Experience subscale items (Section D – Transplant) were analyzed as independent items due to poor internal consistency (Cronbach’s alpha for all 5 items = .38). Based on this evaluation and concerns about construct validity, 2 items were removed from the HIV+ Transplant Experience subscale. Specifically, item D1, “My recovery was harder than I imagined” was removed since its connection to ethics concerns was unclear; and item D3, “Because of my race, accepting this organ was the best chance I had at getting a transplant” because it’s hypothesized relationship would only be true for racial minority respondents and uninterpretable for others.

**Table 4:** Internal consistency reliability; distribution of scales, items, and summary ethics score; and construct validity scores

	Cronbach’s Alpha	mean	SD	Median	IQR	Range
A. Emotions (a1-a8)	0.9357	13.5	6.0	12.5	8–19.5	8–26
B. Trust (b1-b8)	0.8889	17.4	6.6	17	12.5–23	8–30
C. Decision (c1-c3)	0.8432	4.8	1.9	4.5	3–6	3–10
E. Satisfaction (e1-e5)	0.9530	7.9	4.7	6.5	5–9	5–25
D. Transplant (d1-d5)^a^	0.3834					
Transplant_d2	–	1.55	0.6	1.5	1–2	1–3
Transplant_d4	–	2.55	1.3	3	1–3	1–5
Transplant_d5	–	2.35	1.0	2	1.5–3	1–4
Summary ethics score^b^	–	0.85	1.1	0	0–2	0–3
PCS^c^	–	45.6	10.8	43.4	39.1–53.8	14.3–57.2
MCS^c^	–	55.1	9.0	56.7	53.2–59.3	28.1–71.3

^a^Following an initial analysis, two items were removed from the initial transplant subscale and the remaining items analyzed as individual items.

^b^The summary ethics score was developed from Domain B, C, and E. Participants scored in the upper quartile got 1 point for each domain. The sum of the points from Domain B, C, and E consisted of the overall score.

^c^PCS and MCS scores are the physical and mental component summary scores of the KDQOL-36TM [25]. Standardized combined scores for the United States population were used. *N* = 19, 1 participant missed the answer of question 3c, 6, 7, 8, 9a-i, and 10 was excluded from this analysis

In general, the scale scores are right skewed. Distribution of the Emotions, Trust, Decision, and Satisfaction scales and the Transplantation items are shown in Table 4. Each multi-item scale’s internal consistency coefficient exceeded its correlations with other scales, as well as with the Physical Component Summary (PCS) and Mental Component Summary (MCS) scores of the KDQOL-36™ [25], administered at the 6-months post-transplant visit. PCS was negatively correlated with all multi-item scales. As expected, MCS had a stronger correlation with the Emotions scale (correlation coefficient: − 0.80) and weak correlation with other scales (Table 5).

**Table 5:** Correlation of scales, summary score, and construct validity scores. Cronbach’s alpha for multi-item scales given on the diagonal (in parentheses)

	Emotions	Trust	Decision	Satisfaction	Summary score	PCS
Emotions	(0.9357)					
Trust	−0.1782	(0.8889)				
Decision	0.1847	0.5634	(0.8432)			
Satisfaction	0.1892	0.6227	0.9027	(0.9530)		
Summary score	0.0724	0.7149	0.8505	0.818		
PCS	−0.4785	−0.4357	−0.5852	−0.6936	−0.4885	
MCS	−0.8036	0.4605	0.1161	0.158	0.2734	0.2024

To be able to garner a more comprehensive assessment regarding the ethical aspects of experimental HIV D+/R+ transplantation, we developed an exploratory ethics summary score comprised of the scores of the Trust in Medical Care, Decision Making and Consent, and Decisional Regret subscales. Scoring of particular items was reversed where necessary so that all were uniform in terms of potentially problematic responses. Participants received 1 point for each scale where they scored in the upper quartile. The sum of the points from each scale make up the overall score, with a score of 0 indicating no ethical flag and a score of 3 indicating the highest possible ethical flag. Among the 20 participants, 55% had a score of 0, 15% had a score of 1, 20% had a score of 2, and 10% had a score of 3.

## Discussion

Overall, members of the first cohort of participants enrolled in a large nationwide clinical trial of HIV D+/R+ renal transplantation generally reported positive experiences regarding salient ethics domains related to their participation (Table 4). In particular, 6 months following their experimental transplants, participants commonly  reported favorable experiences with the decision-making and consent process to accept an HIV D+ organ (based on an aggregate score of having enough information, not feeling pressure, and being satisfied with the consent process). Further, recipients tended to be neutral regarding whether accepting the HIV D+ organ provided their best chance of getting a transplant given their HIV+ status, perhaps suggesting that they were not pressured to make an unwelcome choice based on their perception of organ availability and access to a life-sustaining intervention. As a related matter, despite the uncertain risks at the time they gave consent to receive an HIV D+ organ, preliminary data suggest that recipients were generally satisfied with their decision to accept an HIV D+ organ and were doing well emotionally following their transplants. Recipients also reported substantial trust, which is consistent with their having agreed to participate in a study evaluating experimental transplants. Finally, participants tended to agree that their HIV D+/R+ transplant made them feel better about living with HIV. This finding is salutary given the stigma that is associated with HIV.

While each of the ethics domains and selected items in the PROMETHEUS battery provide important insight into the experiences of recipients of experimental HIV D+/R+ transplants, we explored the possibility of creating a preliminary summary ethics score to provide an overarching impression of these novel transplants. Overall, the summary ethics score was sanguine with a median of 0, although 2 participants had a score of 3. Nevertheless, to date, patient-participants have not reported substantial ethics concerns with the conduct of the trial. However, this and the generally positive reported experiences in regard to key ethics domains and items may not be surprising given that participants had survived a life-changing transplant by the time they completed the battery.

Nevertheless, in interpreting these preliminary findings it is important to recognize that these data come from a small number of patient-participants in the context of a single carefully conducted clinical trial. The small sample size unfortunately precludes conducting robust psychometric evaluations of PROMETHEUS, such as exploratory factor analyses. In addition, since the data derive from one high-profile clinical trial, the data may not be generalizable to other settings. Regardless, this is the largest available cohort to date of patients undergoing experimental HIV D+/R+ renal transplants in the United States. In the future, it will be essential to evaluate emerging data from this and other related transplant trials, not only to provide further evidence for the validity of PROMETHEUS through the conduct of proper psychometric analyses, but also to identify any potential ethics concerns that may arise.

As the battery is used in clinical transplantation trials, investigators, Institutional Review Boards, and Data Safety and Monitoring Boards may want to use responses to some or all PROMETHEUS scales or items to trigger action on the part of research teams. To accomplish this, future studies should determine how to interpret scores on PROMETHEUS, including its performance as a screening tool to identify potential ethical issues. Such an approach could provide additional protection of research participants.

## Conclusions

We used a rigorous, patient centered approach to develop PROMETHEUS, a battery of measures to interrogate patients’ experiences concerning the ethical issues associated with this novel field of HIV D+/R+ transplantation. By nesting PROMETHEUS within a large clinical trial, we were able to provide preliminary data on its performance, and also to demonstrate the feasibility of nesting an ethics-related patient measure in such transplantation trials.

## Supplementary Information

**Additional file 1: Appendix.**

## Data Availability

The datasets used and analyzed in developing PROMETHEUS are available from the corresponding author on reasonable request.

## Abbreviations

HIV D+/R +: HIV-positive donor organs for HIV+ recipients
MCS: Mental Component Summary
PCS: Physical Component Summary
PLWH: People living with HIV
PRE: Patient-reported experiences
PRO: Patient-reported outcomes
PROMETHEUS: Patient-Reported Measure of Experimental Transplants with HIV and Ethics in the United States
