# Supplementary material for: Development of a Patient Reported Measure of Experimental Transplants with HIV and Ethics in the United States (PROMETHEUS)
Source: J Patient Rep Outcomes. 2021 Mar 18;5:28. doi: 10.1186/s41687-021-00297-y (PMC7973329; doi:10.1186/s41687-021-00297-y)
Supplement: Supplementary file 1 — Additional file 1: Appendix. [file 41687_2021_297_MOESM1_ESM.docx]

**PROMETHEUS Fielded Battery**

**This survey includes a wide variety of questions related to how you are feeling and the decisions you made regarding the possibility of receiving an HIV+ organ.**

**Directions: Please circle the one response that BEST describes your answer to the question.**

**Section A: Emotions**

**********************************************************************************************

**In the past 7 days...**

| **A1. I felt fearful...** | | | | |
| --- | --- | --- | --- | --- |
| 1) Never | 2) Rarely | 3) Sometimes | 4) Often | 5) Always |
| **A2. I found it hard to focus on anything other than my anxiety...** | | | | |
| 1) Never | 2) Rarely | 3) Sometimes | 4) Often | 5) Always |
| **A3. My worries overwhelmed me...** | | | | |
| 1) Never | 2) Rarely | 3) Sometimes | 4) Often | 5) Always |
| **A4.** **I felt uneasy...** | | | | |
| 1) Never | 2) Rarely | 3) Sometimes | 4) Often | 5) Always |
| **A5. I felt nervous...** | | | | |
| 1) Never | 2) Rarely | 3) Sometimes | 4) Often | 5) Always |
| **A6. I felt like I needed help for my anxiety...** | | | | |
| 1) Never | 2) Rarely | 3) Sometimes | 4) Often | 5) Always |
| **A7. I felt anxious...** | | | | |
| 1) Never | 2) Rarely | 3) Sometimes | 4) Often | 5) Always |
| **A8. I felt tense...** | | | | |
| 1) Never | 2) Rarely | 3) Sometimes | 4) Often | 5) Always |

**Section B: Trust**

******************************************************************************************

| **B1. Sometimes doctors care more about what is convenient for them than about their patients’ medical needs.** | | | | |
| --- | --- | --- | --- | --- |
| 1) Strongly Agree | 2) Agree | 3) Neither Agree nor Disagree | 4) Disagree | 5) Strongly Disagree |
| **B2. Doctors are extremely thorough and careful.** | | | | |
| 1) Strongly Agree | 2) Agree | 3) Neither Agree nor Disagree | 4) Disagree | 5) Strongly Disagree |
| **B3. You completely trust doctors’ decisions about which medical treatments are best.** | | | | |
| 1) Strongly Agree | 2) Agree | 3) Neither Agree nor Disagree | 4) Disagree | 5) Strongly Disagree |
| **B4. A doctor would never mislead you about anything.** | | | | |
| 1) Strongly Agree | 2) Agree | 3) Neither Agree nor Disagree | 4) Disagree | 5) Strongly Disagree |
| **B5. All in all, you trust doctors completely.** | | | | |
| 1) Strongly Agree | 2) Agree | 3) Neither Agree nor Disagree | 4) Disagree | 5) Strongly Disagree |
| **B6. The health care system experiments on patients without them knowing.** | | | | |
| 1) Strongly Agree | 2) Agree | 3) Neither Agree nor Disagree | 4) Disagree | 5) Strongly Disagree |
| **B7. The health care system does its best to make patients’ health better.** | | | | |
| 1) Strongly Agree | 2) Agree | 3) Neither Agree nor Disagree | 4) Disagree | 5) Strongly Disagree |
| **B8. Patients get the same medical treatment from the health care system, no matter what the patient’s race or ethnicity.** | | | | |
| 1) Strongly Agree | 2) Agree | 3) Neither Agree nor Disagree | 4) Disagree | 5) Strongly Disagree |

**Section C: Decision Making**

******************************************************************************************

| **C1. I got all the information I needed to make a good decision about getting the new organ.** | | | | |
| --- | --- | --- | --- | --- |
| 1) Strongly Agree | 2) Agree | 3) Neither Agree nor Disagree | 4) Disagree | 5) Strongly Disagree |
| **C2. I felt pressure to accept the new organ.** | | | | |
| 1) Strongly Agree | 2) Agree | 3) Neither Agree nor Disagree | 4) Disagree | 5) Strongly Disagree |
| **C3. I was satisfied with the consent process to get the new organ.** | | | | |
| 1) Strongly Agree | 2) Agree | 3) Neither Agree nor Disagree | 4) Disagree | 5) Strongly Disagree |

**Section D: Transplant**

******************************************************************************************

| **D1. My recovery was harder than I imagined.** | | | | |
| --- | --- | --- | --- | --- |
| 1) Strongly Agree | 2) Agree | 3) Neither Agree nor Disagree | 4) Disagree | 5) Strongly Disagree |
| **D2. Trust in doctors and nurses who took care of me was a significant factor in my decision to get a new organ.** | | | | |
| 1) Strongly Agree | 2) Agree | 3) Neither Agree nor Disagree | 4) Disagree | 5) Strongly Disagree |
| **D3. Because of my race, accepting this organ was the best chance I had at getting a transplant.** | | | | |
| 1) Strongly Agree | 2) Agree | 3) Neither Agree nor Disagree | 4) Disagree | 5) Strongly Disagree |
| **D4. Because of my HIV status, accepting this organ was the best chance I had at getting a transplant.** | | | | |
| 1) Strongly Agree | 2) Agree | 3) Neither Agree nor Disagree | 4) Disagree | 5) Strongly Disagree |
| **D5. Being able to get this transplant made me feel better about having HIV*.*** | | | | |
| 1) Strongly Agree | 2) Agree | 3) Neither Agree nor Disagree | 4) Disagree | 5) Strongly Disagree |

**Section E: Decision Satisfaction**

**The next set of questions are about your decision to get the new organ.**

******************************************************************************************

| **E1. It was the right decision.** | | | | |
| --- | --- | --- | --- | --- |
| 1) Strongly Agree | 2) Agree | 3) Neither Agree nor Disagree | 4) Disagree | 5) Strongly Disagree |
| **E2. I regret the decision that was made.** | | | | |
| 1) Strongly Agree | 2) Agree | 3) Neither Agree nor Disagree | 4) Disagree | 5) Strongly Disagree |
| **E3. I would make the same decision if I had to do it again.** | | | | |
| 1) Strongly Agree | 2) Agree | 3) Neither Agree nor Disagree | 4) Disagree | 5) Strongly Disagree |
| **E4. The decision did me a lot of harm.** | | | | |
| 1) Strongly Agree | 2) Agree | 3) Neither Agree nor Disagree | 4) Disagree | 5) Strongly Disagree |
| **E5. The decision was a wise one.** | | | | |
| 1) Strongly Agree | 2) Agree | 3) Neither Agree nor Disagree | 4) Disagree | 5) Strongly Disagree |
